# Aldehyde dehydrogenase 1 expression is correlated with poor prognosis in breast cancer

**DOI:** 10.1097/MD.0000000000007171

**Published:** 2017-06-23

**Authors:** Juan Yao, Qin Jin, Xu-dong Wang, Hui-jun Zhu, Qi-chao Ni

**Affiliations:** a Department of Pathology, Affiliated Hospital of Nantong University, Nantong; b Department of Pathology, Huaiyin Hospital of Huai’an city, Huai’an; c Department of Laboratory Medicine; d Department of Clinical Tissue Bank; e Department of General Surgery, Affiliated Hospital of Nantong University, Nantong, China.

**Keywords:** ALDH1, breast cancer, clinicopathological factors, immunohistochemistry, prognosis, qRT-PCR

## Abstract

Breast cancer (BC) is one of the most common cancers worldwide, and is a major cause of death in women. Aldehyde dehydrogenase 1 (ALDH1) is a marker of stem cells and cancer stem cells, and its activity correlates with the outcome of various tumors, including BC. This study aimed to analyze the relationship between ALDH1 expression and clinicopathological characters in BC and the prognostic significance of ALDH1.

We used quantitative reverse-transcription PCR (qRT-PCR) to detect *ALDHA1* mRNA levels in 25 fresh frozen BC samples and matched noncancerous samples. Immunohistochemistry on tissue microarrays was used to analyze protein expression in 137 paraffin-embedded BC tissues and corresponding noncancerous tissues. STATA 16.0 software was used for statistical analysis.

The results suggested that levels of both ALDH1 mRNA and protein in BC were significantly higher than in corresponding adjacent breast samples (3.856 ± 0.3442 vs 1.385 ± 0.1534, *P* < .001; 52.6% vs 25.5%, *P* < .001, respectively). ALDH1 protein expression was also significantly associated with histological grade (*P*  =  .017), tumor size (*P*  =  .017), and tumor–node–metastasis (TNM) stage (*P*  =  .038). Multivariate analysis using the Cox regression model demonstrated that ALDH1 expression (*P*  =  .024), molecular typing (*P*  =  .046), and TNM classification (*P*  =  .034) were independent predictive factors for the outcome of BC. Kaplan–Meier analysis and the log-rank test indicated that patients with high ALDH1 expression, triple-negative BC, and advanced TNM stage had a reduced overall survival time.

These data suggest that ALDH1 could be used as a prognostic factor for BC and may provide a useful therapeutic target in the treatment of BC.

## 1 Introduction

Breast cancer (BC) is one of the most common cancer types that has recently become one of the major causes of female deaths both in the United States (USA) and China. Around 246,660 estimated new cases and 40,450 estimated deaths from BC were reported in the USA in 2016.^[1]^ In China, BC is the most common cancer among women, with around 268,000 new cases reported in 2015, representing 15% of all new cancer cases.^[2]^ Although many measures have been implemented for its early detection and diagnosis, mortality levels remain high. Indeed, in 2015, around 69,500 deaths occurred in China from BC recurrence and metastasis. This might be because current therapy only destroys differentiated cells, not drug-resistant tumor cells such as breast cancer stem cells (BCSCs) which can self-replicate and proliferate. BCSCs are often in a dormant stage (G0/G1) during drug treatment, and new tumor cells produced after treatment cause BC recurrence. BCSCs can also promote metastasis, heterogeneity, and therapeutic resistance.^[3]^ Therefore, it is important to develop novel targets and treatment strategies for BC.

Aldehyde dehydrogenase 1 (ALDH1) is a detoxifying enzyme that oxidizes aldehyde into carboxylic acid and converts retinol into retinoic acid. It is ubiquitously distributed in various tumors, including BC, nonsmall-cell lung cancer, laryngeal cancer, ovarian cancer, and gastric cancer.^[4–8]^ Many studies showed that ALDH1 is a marker of BCSCs,^[3,5,9,10]^ while Kunju et al^[11]^ reported that ALDH1-positive benign breast epithelial cells may predict an increased risk for the development of BC. Several studies suggested that ALDH1 is associated with an aggressive phenotype and poor prognosis,^[12–16]^ although others found that it was not a survival predictor of BC^[17–19]^; another study reported that expression of the major ALDH1 isoenzyme ALDH1A1 predicted a better outcome in BC of the triple-negative subtype.^[20]^ The prognostic value of ALDH1 in BC is therefore controversial, and has not been fully elucidated. In the present study, we explored the relationship between ALDH1 mRNA and protein expression and clinicopathological characteristics of BC patients, and evaluate its prognostic significance in BC.

## 2 Materials and methods

### 2.1 Sample collection

We collected 25 fresh frozen BC samples and matched noncancerous samples and 137 paraffin-embedded BC tissue and corresponding noncancerous tissue samples from patients who underwent modified radical mastectomy for BC from January 2002 to May 2010 in the pathology department of the Affiliated Hospital of Nantong University. All patients were followed up for at least 5 years. Patients did not receive any preoperative treatments and had no evidence of distant metastasis. The BC diagnosis was certified by 2 pathologists in our department. The histological stage was classified into I–III according to the Nottingham modified Bloom and Richardson system.^[21]^ Only patients in TNM stages I–III were included in the study. BC was divided into 4 molecular subtypes based on the 2013 St Gallen consensus^[22]^: Luminal A, Luminal B, human epidermal growth factor receptor (HER)-2 overexpression, and triple-negative. Requisite original clinical data were acquired from pathology reports and hospital records, including patient age, tumor size (T stage), lymph node metastasis (N stage), TNM stage, histological grade, hormone receptor (estrogen receptor/progesterone receptor (ER/PR)) status, and HER-2 expression.

### 2.2 Ethics statement

This research was approved by the Ethics Committee of Nantong University. All patients had provided their written consent to participate in this research.

### 2.3 One-step quantitative reverse transcription (qRT-PCR) analysis of fresh frozen BC tissues

Total mRNA was extracted from the 25 fresh frozen BC samples and matched adjacent breast tissues using TRIzol reagent. Primers to amplify *ALDH1A1* were designed using Primer Express Software as follows: forward primer 5′-ACTTACCTGTCCTACTCA-3′, reverse primer 5′-CTTATCTCCTTCTTCTACCT-3′. One-step qRT-PCR was conducted on an ABI 7500 thermal cycler (Applied Biosystems is in America) with an initial denaturation at 95°C for 2 minutes, then 40 cycles of denaturation at 95°C for 10 seconds, annealing and extension at 55°C for 1 minute, and 72°C for 1 minute. All experiments were performed in triplicate.

### 2.4 Immunohistochemical (IHC) analysis of BC tissue microarrays (TMA)

IHC analysis of 4-μm-thick tissue sections was conducted as previously described.^[23,24]^ The interpretation of ER, PR, HER-2, and Ki-67 was based on our previous reported studies.^[22]^ It was considered to be positive when the cytoplasmic components or the cell membrane showed positive staining for ALDH1 expression. The immunostaining score was calculated by multiplying the percentage and the intensity of positive cells. The cutoff point for a statistically significant ALDH1 expression score was acquired by applying the X-title software program (http://www.tissuearray.org). We then further classified the expression score into 2 groups as follows: low or no expression group (<10 score) and high expression group (≥10 score).

### 2.5 Statistical analysis

The Chi-square test was used to compare the relationship between ALDH1 protein expression and clinicopathologic attributes, while Cox proportional hazards regression models were conducted for univariate and multivariate analyses to determine which factor was independently associated with overall survival (OS) and to calculate hazard ratios and 95% confidence intervals. We used Kaplan–Meier analysis and the log-rank test to estimate OS and to determine the difference between OS curves, and to calculate the log-rank *P*-value. *P* < .05 was considered to be statistically significant for all tests. All data were analyzed using STATA 16.0 software.

## 3 Results

### 3.1 *ALDH1A1* mRNA levels in BC and matched noncancerous breast samples

**Figure 1:**
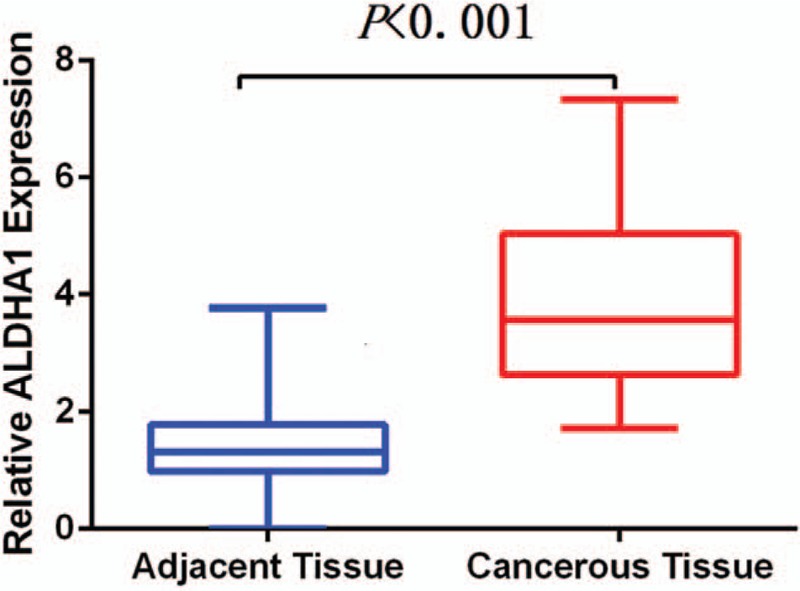
Comparison of ALDH1A1 mRNA level in BC and adjacent breast tissue.

*ALDH1A1* mRNA levels were assessed by qRT-PCR, and shown to be significantly higher in BC samples than in corresponding adjacent breast samples (3.856 ± 0.3442 vs 1.385 ± 0.1534, *P* < .001) (Fig. 1).

### 3.2 ALDH1 protein expression in BC and matched noncancerous samples

**Figure 2:**
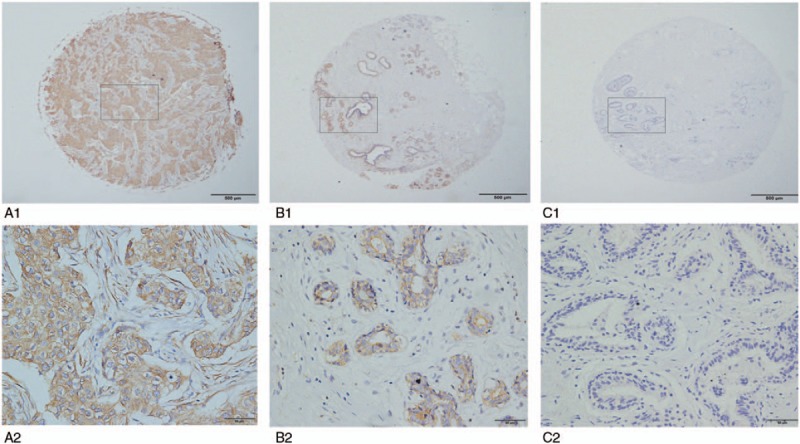
Expression of ALDH1 protein in BC and adjacent breast samples: high expression in BC (A1 × 40, A2 × 400); high expression in adjacent breast tissue (B1 × 40, B2 × 400); no expression in adjacent breast tissue (C1 × 40, C2 × 400).

We next evaluated ALDH1 protein expression in the BC TMA. ALDH1 was mainly expressed in the cytoplasm of breast cells (Fig. 2). High ALDH1 expression was seen in 52.6% (72/137) BC samples compared with 25.5% (35/137) noncancerous breast tissue samples. This difference was statistically significant (*χ*^2^  =  20.992, *P* < .001).

### 3.3 Correlations between ALDH1 protein expression and clinicopathological factors

We investigated the relationship between ALDH1 protein expression and important clinical characteristics (Table 1). Significant correlations were identified between ALDH1 protein expression and histological grade (*P*  =  .017), tumor size (*P*  =  .017), and TNM stage (*P*  =  .038), but not with other variables, including age, molecular classification, and lymph node metastasis.

**Table 1:**
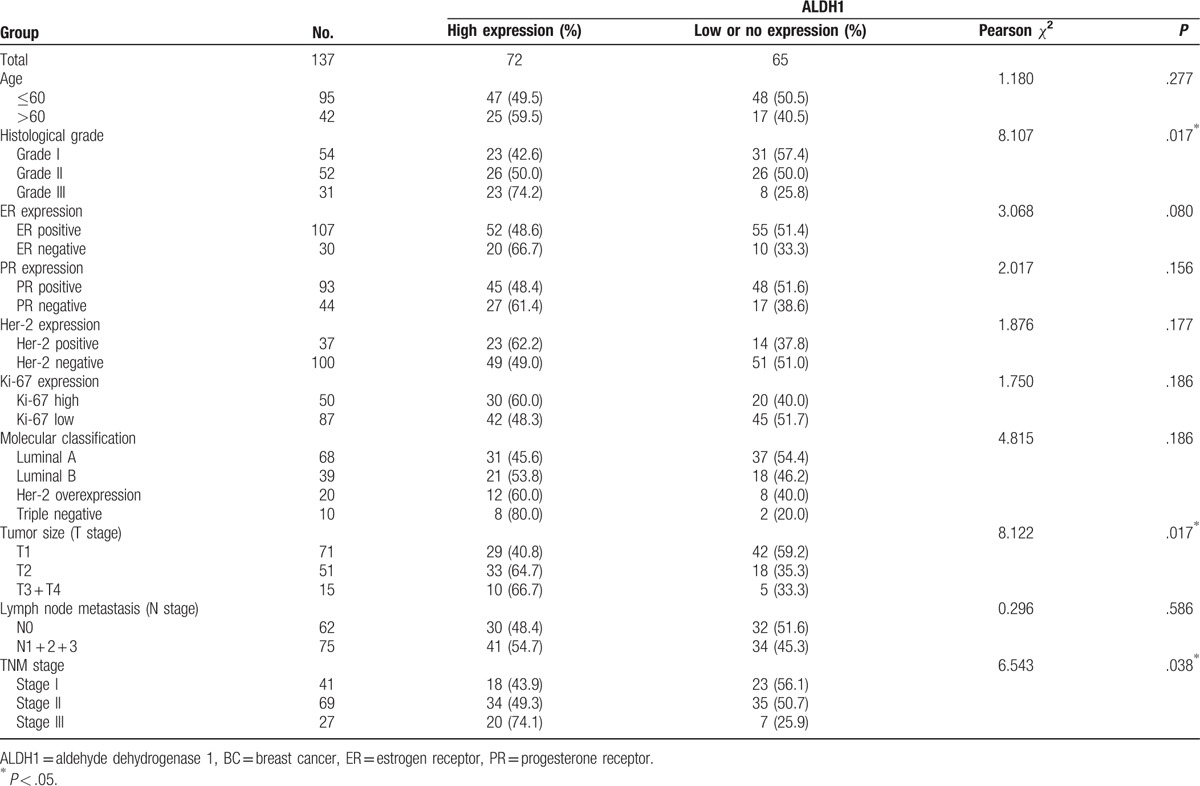
Correlation of ALDH1 expression with clinical parameters in BC.

### 3.4 Survival analysis

**Figure 3:**
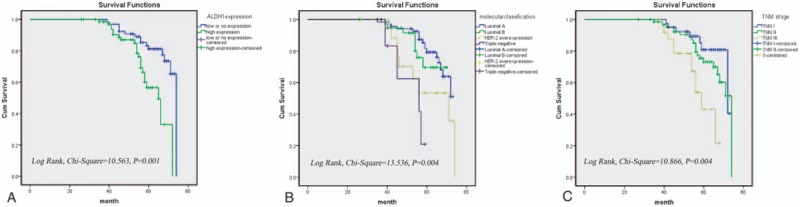
Five-year survival curve of ALDH1 expression (A), molecular classification (B), and TNM stage (C) in BC.

Univariate analysis showed that the OS of 137 BC patients was associated with ALDH1 expression levels, molecular classification, histological grade, and TNM stage. Multivariate analysis showed that only ALDH1 expression, molecular classification, and TNM stage were independent prognostic factors for OS (Table 2). Moreover, survival curves constructed from Kaplan–Meier analysis indicated that high ALDH1 levels, poor molecular classification (HER-2 overexpression and the triple-negative subtypes), and an advanced TNM stage all had an unfavorable effect on OS (Fig. 3).

**Table 2:**
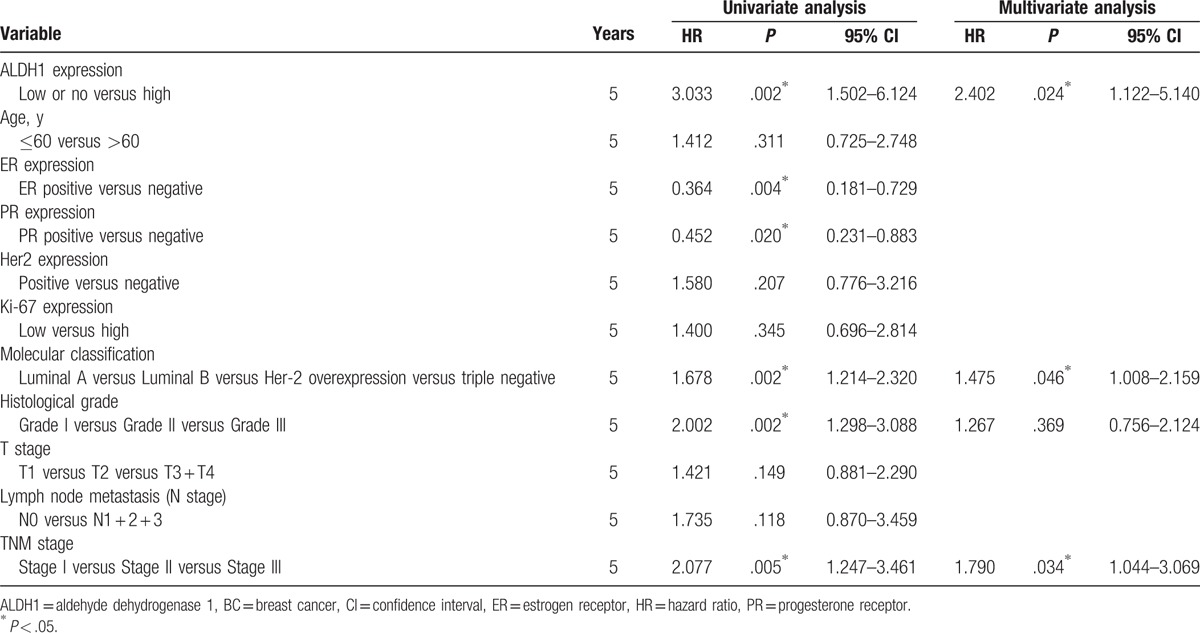
Univariate and multivariate analysis of prognostic factors in BC for overall survival.

## 4 Discussion

A number of studies have indicated that CSCs contribute to tumor initiation, development, metastasis, and recurrence^[25,26]^ while some research also showed that they play a role in drug resistance.^[12,27]^ CSC markers were reported to be present in CSCs as well as in normal stem cells,^[9]^ with ALDH1 shown to be a marker of BCSCs. As BC is one of the most common tumors in women worldwide, so we conduct this study to explore the role of ALDH1 expression in BC. In the present study, we provide important insights into the role of ALDH1 mRNA and protein in BC. We found that *ALDH1A1* mRNA levels were significantly higher in BC than in adjacent breast tissues, and similarly that ALDH1 protein expression was significantly higher in BC than in matched noncancerous samples.

Previous studies indicated that ALDH1 positivity accounted for only a small percentage of BC cases, ranging from 8.4% to 24.8%,^[12,28,29]^ whereas Pan et al^[14]^ reported that this percentage could be as high as 93%. This compares with our present study, which detected a frequency of 52.6%. This broad range may be attributable to differences in cutoff points and sample selection. For example, a study by Ricardo et al^[30]^ observed that ALDH1 protein expression was variable in the 4 molecular subtypes: Luminal A (5.1%), Luminal B (12.2%), HER-2 overexpression (12.29%), and triple-negative (25%). In our study, these corresponding rates were 45.6%, 53.8%, 60.0%, and 80%, respectively. These data suggest that ALDH1 is more highly expressed in the triple-negative subtype than the luminal subtypes, which enables us to better understand the poor outcome of triple-negative BC.

Mansour and coworkers^[10,12,31]^ previously reported that ALDH1 was associated with lymph node metastasis and tumor size, while Pan et al^[14]^ similarly showed that ALDH1 was correlated with tumor size as well as histological grade and Ki67 expression. Consistent with these studies, we observed significant correlations between ALDH1 and some aggressive attributes, including tumor size, high histological grade, and advanced TNM stage. It was also more commonly detected in association with lymph node metastasis and the triple-negative subtype, as reported by Lee et al^[32]^ and Park et al,^[33]^ although these associations were not significant. Multivariate analysis indicated that patients with high ALDH1 expression had a shorter survival time compared with those with low ALDH1 expression, which is consistent with previous reports.^[12,13,16]^ These findings indicate that ALDH1 is an aggressive characteristic of BC, which might reflect the self-renewal and differentiation potential of ALDH1+ BC. Moreover, ALDH1 appears to have a functional role in the detoxification of drugs that are commonly used to treat cancer, as well as in the modulation of cell proliferation, and the Notch signaling pathway.^[34,35]^

This study has a number of limitations. First, the TMA used for IHC may contribute to the limitation of analyzing only a small number of stem cells. Therefore, larger samples are necessary to confirm our findings in the future. Second, we used archived paraffin-embedded samples for convenience, which could introduce bias to this retrospective observational study. Third, patients with different types of postoperative therapy might affect the prognostic analysis, so additional prospective case–control studies should be conducted to verify our results.

In conclusion, our results suggest that ALDH1 expression correlates with aggressive phenotypes, and that high ALDH1 expression predicts a poor outcome in BC. Thus, ALDH1 could be used as a potent prognostic marker for patients with invasive ductal carcinoma. Further research is required to analyze the role of ALDH1 in the development of BC and to clarify its mechanism.

## Acknowledgment

We would like to thank the patients, doctors, and graduate students who participated in this study.
